# Comparison of Double-Stranded DNA at the 5′ and 3′ Ends of the G-Triplex and Its Application in the Detection of Hg(II)

**DOI:** 10.3390/ijms25158159

**Published:** 2024-07-26

**Authors:** Yule Cai, Ziyi Wu, Xiangxiang Li, Xingting Hu, Jiamin Wu, Zhengying You, Jieqiong Qiu

**Affiliations:** College of Life Sciences and Medicine, Zhejiang Sci-Tech University, Hangzhou 310018, China

**Keywords:** Hg(II) detection, G-triplex, thioflavin T, label-free, fluorescence

## Abstract

Leveraging the fluorescence enhancement effect of the G-triplex (G3)/thioflavin T (ThT) catalyzed by the adjacent double-stranded DNA positioned at the 5′ terminus of the G3, the G3-specific oligonucleotide (G3MB6) was utilized to facilitate the rapid detection of mercury (Hg(II)) through thymine–Hg(II)–thymine (T-Hg(II)-T) interactions. G3MB6 adopted a hairpin structure in which partially complementary strands could be disrupted with the presence of Hg(II). It prompted the formation of double-stranded DNA by T-Hg(II)-T, inducing the unbound single strand of G3MB6 to spontaneously form a parallel G3 structure, producing a solid fluorescence signal by ThT. Conversely, fluorescence was absent without Hg(II), since no double strand and formation of G3 occurred. The fluorescence intensity of G3MB6 exhibited a positive correlation with Hg(II) concentrations from 17.72 to 300 nM (R^2^ = 0.9954), boasting a notably low quality of limitation (LOQ) of 17.72 nM. Additionally, it demonstrated remarkable selectivity for detecting Hg(II). Upon application to detect Hg(II) in milk samples, the recovery rates went from 100.3% to 103.2%.

## 1. Introduction

Mercury (Hg(II)), a naturally occurring heavy metal, poses significant toxicity risks and can accumulate and migrate within environmental matrices [1]. Beyond natural processes such as volcanic eruptions, geothermal activities, and forest fires, Hg(II) is a byproduct of various industrial activities. Hg(II) in gaseous or liquid forms is produced during mineral mining and the disposal of waste products, including cement, pesticides, and lamps. Hg(II) disperses into the air, water, and soil, subsequently entering the human body through bioaccumulation, and can result in severe damage to the liver, brain, and nervous system, posing serious health risks [2]. According to the World Health Organization (WHO), the allowable concentrations of Hg(II) in potable water must be below 1 ppb (10^−9^, equal to μg/L) [3]. Consequently, detecting trace levels of Hg(II) in the environment is critical for safeguarding human health. Traditional techniques of detection, including selective photoelectrochemical (PEC) methods [4], anodic stripping voltammetry (ASV) [5], inductively coupled plasma-mass spectrometry (ICP-MS) [6,7], fluorescence spectrometry [8,9,10], colorimetric methods [11], and enzyme-linked immunosorbent assays [12], offer high accuracy and sensitivity. However, their practical application is hindered by the complex operational requirements and substantial testing costs. Specifically, ICP-MS encounters higher detection errors at lower Hg(II) concentrations and necessitates specialized personnel. Thus, an urgent demand is for developing more straightforward and direct techniques of detection for Hg(II).

In recent years, functional nucleic acids, specifically ligand binders known as aptamers, have gained much attention in the construction of biosensors because of their advantages of facile modification, low costs of synthesis, and high sensitivity [13]. It is well established that Hg(II) can interact with the N3 positions of the adjacent thymine (T) bases, replacing the imino proton to form the T-Hg(II)-T mismatched pairs [14]. Consequently, DNA sequences rich in T bases can form T-Hg(II)-T complexes, making them suitable candidates for use as Hg(II)-specific aptamers. For example, the T-T mismatch in double-stranded DNA (dsDNA) can selectively and tightly bind Hg(II), forming a T-Hg(II)-T complex. The Hg(II)-mediated dsDNA structure often exhibits more excellent structural stability than conventional AT/TA base pairing [15]. As a result, T-rich oligonucleotides are frequently utilized in constructing biosensors as Hg(II)-specific aptamers. Notably, Hg(II) can induce conformational changes in its DNA aptamers, leading to the formation of secondary structures such as hairpins [16], DNA duplexes [17], and G-quadruplexes [18]. These structural transformations can be detected using fluorescent intercalating dyes designed to recognize such changes. In biosensor designs, Hg(II) can act as a fluorescent switch by using T-rich dsDNA in conjunction with fluorescent dyes such as 4′,6-diamidino-2-phenylindole (DAPI) [16], thioflavin T (ThT) [19,20], and SYBR Green I [21]. The conformational changes in Hg(II)-specific aptamers induced by Hg(II) binding alter the fluorescence signal, which can be measured precisely.

Additionally, the G-triplex (G3) is acknowledged as a distinctive secondary DNA structure capable of effective monitoring by ThT [22]. ThT preferentially binds to a parallel G3 structure, producing a more powerful fluorescence signal than an antiparallel or mixed parallel–antiparallel structure. It offers a more flexible and adaptable DNA configuration, thus enhancing the potential for the development of Hg(II) biosensors. The versatility of the DNA structure opens numerous possibilities for the innovative design and optimization of biosensors. Wang et al. designed intramolecular dsDNA structures using T-Hg(II)-T mismatched complexes to facilitate the detection of Hg(II) [23]. The design, which places dsDNA adjacent to the G3 structure, significantly enhanced the fluorescence signal of the G3/ThT complex. Moreover, analysis of the G3 folding dynamics indicated that the 5′ overhangs of G3 preferentially promote the formation of a parallel G3 structure over an antiparallel one [24]. In other words, the proximal DNA to the 5′ or 3′ ends of the G3 structure can differentially affect its conformation. However, the effect of the dsDNA at the 5′ and 3′ ends of G3 on G3’s folding still needs to be fully understood.

The study designed a series of oligonucleotides, as shown in Table S1, to monitor the folding of G3 induced by the adjacent dsDNA at the 5′ or 3′ end of G3 using ThT. Specifically, the Hg(II) aptamers G3MB6 contains single-stranded DNA (ssDNA) with the sequence of 5′-TGCTTAGTCCCTAGCTATATGGGAAGGGAGGG-3′, which includes the G3 motif. cDNA-9, with the sequence 5′-TAGCTTGGGTCTTTGCA-3′, was constructed to develop a labeling-free method for detecting Hg(II) based on the formation of a G3-ThT complex, thereby improving the sensitivity of detection. Without Hg(II), G3MB6 forms a DNA hairpin structure that restricts the intercalation of ThT, resulting in no fluorescence signal. With Hg(II), G3MB6 and cDNA-9 form dsDNA through T-Hg(II)-T base pairs, forming the G3 structure. It facilitates the embedding of ThT in the G3, producing a stronger fluorescence signal and enabling the specific detection of Hg(II). Therefore, it offers the advantages of simple operation, good stability, and low cost, providing a novel approach to monitoring trace levels of Hg(II) in natural samples and ensuring human health and safety.

## 2. Results and Discussion

### 2.1. Principles of Detecting Hg(II)

**Scheme 1:**
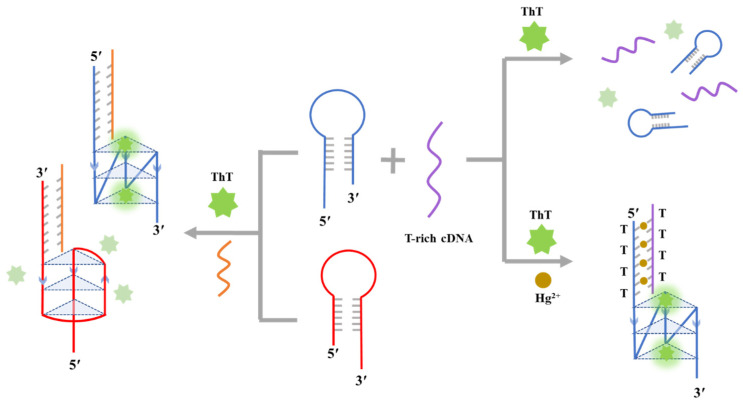
The effect of the adjacent 5′ or 3′ ends of the dsDNA of G3 monitored by ThT and the principles of detecting Mercury (Hg(II)).

ThT is recognized for its ability to form a highly fluorescent G3/ThT complex with parallel G3 structures [22]. As detailed in Scheme 1, the formation of dsDNA at either the 5′ or 3′ end of G3 can influence its folding pattern, thereby modulating the fluorescence signal of the G3/ThT complex. Consequently, the conformation of G3 can be inferred from the specific fluorescence intensity of the G3/ThT complex. Our findings indicate that dsDNA at the 5′ end of G3 enhanced the G3/ThT complex’s fluorescence intensity, suggesting that dsDNA at the 5′ end promotes the formation of parallel G3 structures. In this work, a label-free DNA probe mediated by G3-dsDNA for detecting Hg(II) was developed. A guanine-rich DNA probe, G3MB6, was designed, and the single-stranded cDNA-9 was partially complementary to the 5′ end of G3MB6. Without Hg(II), G3MB6 naturally adopted a hairpin conformation due to being self-complementary, and cDNA-9 remained in a free coil state, resulting in weak fluorescence signals when ThT was added. When the Hg(II) was introduced to the detection system, the T-Hg(II)-T mismatches disrupted the hairpin structure of G3MB6, allowing the 5′ end of G3MB6 to hybridize with cDNA-9, forming a double-stranded region. The hybridization of T-Hg(II)-T further influenced the folding of G3MB6 into a parallel G3 structure, which is consistent with the 5′ overhangs of G3 [24], significantly restricting the rotation of the aromatic ring of ThT and generating a solid fluorescent signal. This enabled the quantitative monitoring of Hg(II) by activating a change in the fluorescent signal. Our investigation into G3’s conformation revealed that dsDNA at different positions (5′ and 3′ ends) directly impacted the folding of G3, which can be monitored by the fluorescence of ThT. Specifically, G3 transitions from a parallel to an antiparallel fold when the dsDNA is positioned at the 3′ terminus, causing substantially lower fluorescence intensity than the configuration with dsDNA at the 5′ end (5′-dsDNA-G3-3′).

### 2.2. Effect of 5′/3′-dsDNA on the Fluorescence Signal of G3/ThT

The reaction’s feasibility was initially optimized to establish the ideal assay conditions. As depicted in Figure S1A, various final concentrations of ThT, ranging from 2 to 8 μM, were detected to determine the concentration that yielded the highest fluorescence intensity (F/F0, where F denotes the fluorescence intensity of G3MB1 + cDNA-1 + ThT and F0 denotes the fluorescence intensity of G3MB1 + ThT). This indicated that the optimal F/F0 was achieved at a concentration of ThT of 4 μM. In determining the optimal reaction time for the formation of a duplex between G3MB1 and cDNA-1, the fluorescence intensity increased along with an increase in the reaction time from 2 to 60 min. Figure S1B illustrates that F/F0 reached a maximum after 60 min, maintaining stability. Subsequently, the detection conditions were refined, particularly for the pH of incubation and the concentrations of ions (Na(I) and Mg(II)) in the 50 mM Tris-HCl buffer. Figure S2A demonstrates that a pH of 7.6 was optimal. The effect of the concentration of salt on F/F0 was also evaluated, with the Na(I) concentrations set at 0, 50, 100, 150, and 200 mM, and the Mg(II) concentrations at 0, 10, 20, 30, and 50 mM. Figure S2B,C show that increasing concentrations of Na(I) and Mg(II) facilitated the formation and stabilization of the DNA’s double helix and G3 structure, thereby enhancing the F/F0 of the detection system. Nevertheless, excessive salt ions in the buffer impeded the system’s performance, with optimal concentrations identified as 100 mM for Na(I) and 20 mM for Mg(II). Lastly, the reaction temperature of G3MB1 + cDNA-1 and the ThT incubation time were assessed. Reaction temperatures of 25, 37, 50, and 60 °C were tested. Figure S3A reveals that samples incubated at 37 °C exhibited the highest F/F0 ratio. Elevated temperatures adversely affected the stability and formation of the duplex and the G3 structure, resulting in decreased F/F0. The incubation time of ThT also significantly influenced the fluorescence intensity. Figure S3B indicates that among the five groups (ThT incubated for 1, 5, 10, 20, and 30 min), an incubation time of 5 min was optimal.

**Figure 1:**
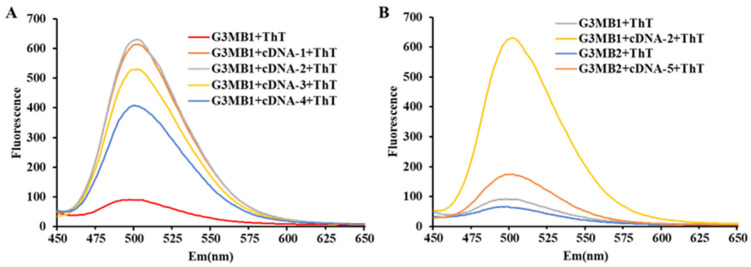
(**A**) Enhancement of the fluorescence of the G3/ThT complex with varying base gaps. (**B**) Impact of G3’s position on the fluorescence signal. For this, 125 nM G3MB1/G3MB2 and 150 nM cDNA-1–cDNA-5, respectively, were reacted in a 50 mM Tris-HCl buffer (pH = 7.6) including 50 mM KCl, 100 mM NaCl, and 20 mM MgCl_2_ at 37 °C for 60 min. Subsequently, a final concentration of 4 μM of ThT was added and reacted for 5 min at 25 °C. The fluorescence signal was detected at *Ex* = 430 nm.

The fluorescence signal of the G3/ThT complex can be significantly affected by the adjacent dsDNA. To investigate the impact of the number of base gaps connecting the adjacent dsDNA to G3, as well as the effects of G3’s conformations on the fluorescence of G3/ThT, we designed G3MB1 and its complementary strands of cDNA-1–4, as detailed in Table S1, with base gaps of 3, 2, 1, and 0, respectively. This demonstrated that the enhancement of the fluorescence of the G3/ThT complex was most pronounced when the number of base gaps was two or three. Conversely, shorter base gaps diminished the fluorescence signals, as illustrated in Figure 1A. We hypothesized that a gap of two or three bases between the adjacent dsDNA and the G3 structure facilitated the formation of a parallel G3 configuration, thereby increasing the binding affinity of ThT. In contrast, a base gap of zero or one may induce a less favorable parallel form in the G3 structure, and the fluorescence intensity of ThT was reduced.

Furthermore, the experiments assessed the effect of the G3’s position on the fluorescence signal. We observed a significant enhancement of the fluorescence signal when the G3 structure was positioned at the 3′ terminus of the DNA strand, indicating that the adjacent dsDNA located at the 5′ terminus of G3, such as in G3MB1, substantially increased the fluorescence signal. In contrast, only a modest enhancement was detected for G3MB2, where the G3 structure was located at the 5′ end. The observed phenomenon was attributed to the formation of the favorable structure of a parallel G3 at the 3′ terminus of G3MB1. The parallel G-triplex was more predisposed to bind ThT, partially restricting the rotation of the aromatic ring within ThT and ultimately generating a robust fluorescent signal, as shown in Figure 1B.

**Figure 2:**
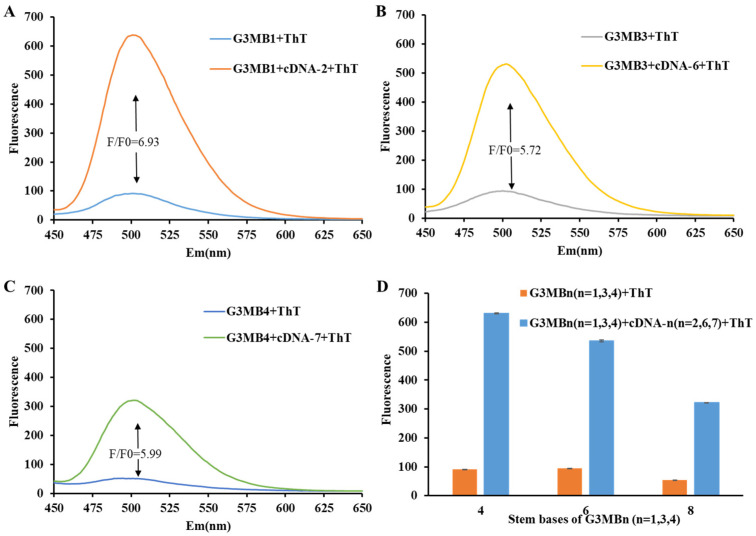
(**A**) Fluorescence intensity spectra of G3MB1 with stem lengths of four base pairs with or without cDNA-2. F denotes the fluorescence intensity of G3MBn (n = 1, 3, 4) + cDNA-n (n = 2, 6, 7) + ThT and F0 denotes the fluorescence intensity of G3MBn (n = 1, 3, 4) + ThT. (**B**) Fluorescence intensity spectra of G3MB3 with stem lengths of six base pairs with or without cDNA-6. (**C**) Fluorescence emission spectra of G3MB4 with stem lengths of eight base pairs with or without cDNA-7. (**D**) Fluorescence intensity of G3MB1, G3MB3, and G3MB4 with stem lengths of four, six, and eight base pairs, respectively, with/without cDNA-2, cDNA-6, and cDNA-7, recorded at *Ex* = 430 nm and *Em* = 505 nm. Each reaction was conducted in a 50 mM Tris-HCl buffer (50 mM KCl + 100 mM NaCl + 20 mM MgCl_2_, pH = 7.6) by adding 125 nM G3MBn (n = 1, 3, 4), 150 nM cDNA-n (n = 2, 6, 7), 4 µM ThT, and 600 nM Hg(II) with a reaction time of 60 min at 37 °C.

The length of its stem directly influences the stability of the hairpin structure. To determine the optimal formation of the hairpin structure, we designed G3MB1, G3MB3, and G3MB4 (Table S1) with stem lengths of four, six, and eight base pairs, respectively. As illustrated in Figure 2, the fluorescence signals of G3MBn (n = 1, 3, 4) were of low intensity, indicating that even a stem length of four base pairs was sufficient to inhibit the formation of the G3. Upon adding their corresponding complementary strands, G3MB1, with a stem length of four base pairs, exhibited the strongest fluorescence signal compared with G3MB3 and G3MB4, which have six and eight base pairs, respectively. As shown in Figure 2D, the fluorescence signals decreased gradually with the increase in the stem length of the hairpin structures. This suggested that shorter stem lengths facilitate more manageable disruption, thereby making the formation of the G3 more controllable. Short stems do not require extensive sequences for recognition, thus shortening the response time.

**Figure 3:**
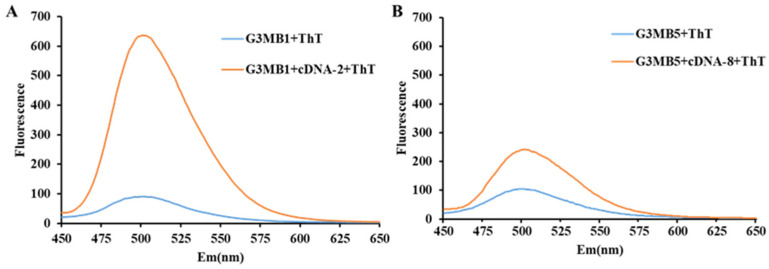
(**A**) Fluorescence intensity spectra of G3MB1 with and without cDNA-2. (**B**) Fluorescence intensity spectra of G3MB5 with and without cDNA-8. Each reaction was conducted in a 50 mM Tris-HCl buffer (50 mM KCl + 100 mM NaCl + 20 mM MgCl_2_, pH = 7.6) by adding 125 nM G3MBn (n = 1, 5), 150 nM cDNA-n (n = 2, 8), 4 µM ThT, and 600 nM Hg(II) with a reaction time of 60 min at 37 °C.

According to prior research, ThT binds to guanine–adenine (GA) sequences in dimeric parallel strands, enhancing fluorescence [25]. Additionally, ThT can recognize guanine–thymine (GT) sequences when forming T-Hg(II)-T mismatched pairs [26]. Our earlier study demonstrated that T-Hg(II)-T mimics thymine–adenine (TA) pairs [16], leading us to hypothesize that GA sequences promote the restriction of ThT’s rotation, increasing its fluorescence. To test this hypothesis, we designed G3MB5 (TGCTACTACCCGAGCTATATGGGTAGGGCGGG) and compared it with G3MB1 (TGCTAAGTCCCGAGCTATATGGGAAGGGAGGG). As shown in Figure 3, G3MB5 exhibited only a weak increase in the fluorescence signal upon binding to cDNA-8. In contrast, G3MB1 displayed a substantial increase in the fluorescence of ThT when paired with cDNA-2. The significant increase in fluorescence can be attributed to the GA sequences in the G3 structure of G3MB1, which likely increased the binding affinity for ThT. Consequently, the structure in G3MB1 demonstrated a more robust fluorescence response, indicating its superior suitability for this detection system.

### 2.3. Feasibility of G3MB6/ThT for Monitoring Hg(II)

The reaction times required for the detection of Hg(II) using G3MB6 and cDNA-9 were investigated to optimize the assay’s efficiency. As depicted in Figure S4A, the longer the reaction time, the higher the relative fluorescence intensity (F/F0), and saturation was reached at 60 min. Beyond this point, extending the reaction time did not further enhance the fluorescence intensity. Additionally, the influence of the reaction temperature on the detection of Hg(II) was evaluated. The highest relative fluorescence intensity (F/F0) was observed at 25 °C, as depicted in Figure S4B. Therefore, it can be concluded that the dsDNA most effectively enhanced the fluorescence signal of the ThT/G3 complex through the T-Hg(II)-T structural linkage when reacted at 25 °C for 60 min. This indicated that the optimal conditions for the detection of Hg(II) used the G3MB6 probe and the incubation temperature of the complementary strand of cDNA-9 was 25 °C, and the reaction time was 60 min.

**Figure 4:**
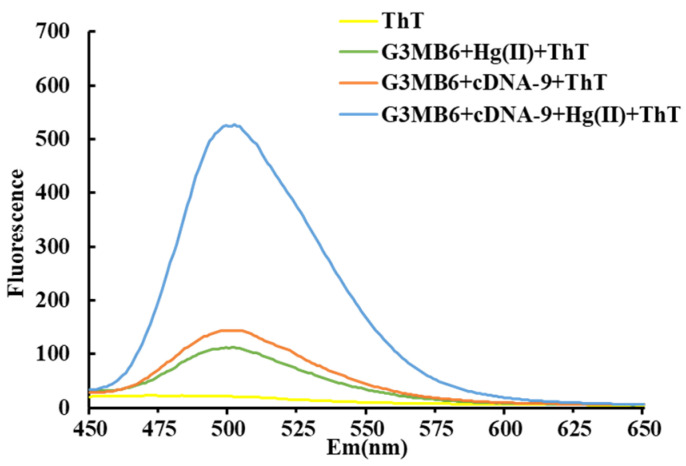
Fluorescence intensity spectra of ThT, G3MB6 + Hg(II) + ThT, G3MB6 + cDNA-9 + ThT, and G3MB6 + Hg(II) + cDNA-9 + ThT. For the reactions, 125 nM G3MB6 and 150 nM cDNA-9 were incubated separately in a 50 mM Tris-HCl buffer (pH = 7.6) containing 50 mM KCl, 100 mM NaCl, and 20 mM MgCl_2_ at 25 °C for 60 min. Subsequently, a final concentration of 4 μM ThT was introduced, and the mixtures were reacted at 25 °C for an additional 5 min, and the fluorescence signal was detected at *Ex* = 430 nm.

To validate the efficacy of this method for detecting Hg(II) ions, we first examined the intrinsic fluorescence signal of ThT in the buffer solution, as shown in Figure 4. In its free state, ThT exhibited minimal fluorescence intensity. Without the complementary strand of cDNA-9, G3MB6 was mixed with Hg(II), and ThT was subsequently added; the fluorescence signal remained low, indicating that the hairpin structure of G3MB6 remained stable in the solution. When Hg(II) was absent, the mixture of G3MB6 and cDNA-9, followed by the addition of ThT, resulted in no significant change in fluorescence compared with G3MB6 alone. This suggested that cDNA-9 alone cannot disrupt the hairpin structure of G3MB6. However, after mixing G3MB6 with cDNA-9 containing Hg(II), the fluorescence signal of ThT was significantly enhanced. This indicated that cDNA-9 can open the hairpin structure of G3MB6 by forming T-Hg(II)-T base pairs with Hg(II). This confirmed the feasibility of the fluorescence method of detecting Hg(II) ions, demonstrating its potential for practical applications in detecting Hg(II) contamination.

### 2.4. Sensitivity and Selectivity of G3MB6/ThT for Detecting Hg(II)

**Figure 5:**
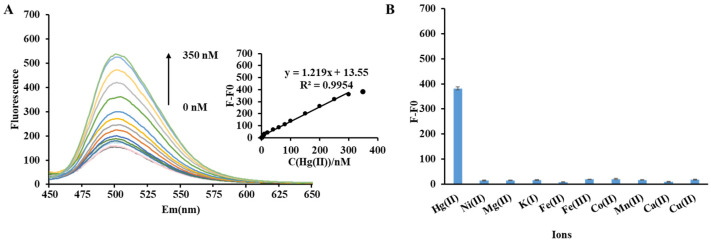
(**A**) Fluorescence intensity spectra of G3MB6 with the concentrations of Hg(II) in the 0–350 nM range at *Ex* = 430 nm. The concentrations of Hg(II) tested were as follows: 0, 2, 4, 6, 8, 10, 20, 40, 60, 80, 100, 150, 200, 250, 300 and 350 nM. The change in fluorescence (F-F0, F indicates the fluorescence signal of G3MB6 + cDNA-9 + ThT with Hg(II) and F0 indicates the fluorescence signal of G3MB6 + cDNA-9 + ThT without Hg(II) at *Em* = 505 nm) showed a linear relationship with the concentration of Hg(II) (0–300 nM). For this, 125 nM G3MB6, 150 nM cDNA-9, and 4 µM ThT were used in a 50 mM Tris-HCl buffer (50 mM KCl + 100 mM NaCl + 20 mM MgCl_2_; pH 7.6). (**B**) Changes in the fluorescence signal (F-F0) of G3MB6 in the presence of Hg(II), Ni(II), Mg(II), K(I), Fe(II), Fe(III), Co(II), Mn(II), Ca(II), and Cu(II) at *Ex* = 430 nm and *Em* = 505 nm. For this 600 nM Hg(II) and 3 µM other metal ions were used; 125 nM G3MB6, 150 nM cDNA-9, and 4 µM ThT were used in a 50 mM Tris-HCl buffer (50 mM KCl + 100 mM NaCl + 20 mM MgCl_2_; pH = 7.6) for each reaction. F indicates the fluorescence signal of G3MB6 + cDNA-9 + ThT with metal ions and F0 indicates the fluorescence signal of G3MB6 + cDNA-9 + ThT without metal ions at *Em* =505 nm.

The sensitivity of the G3MB6/ThT sensor to detect Hg(II) was investigated, and a solution with incrementally increasing concentrations of Hg(II) was introduced into the detection system. As shown in Figure 5A, in the 0–300 nM range, with an increase in the concentration of Hg(II), the fluorescence intensity gradually increased. Without Hg(II), the G3MB6 probe spontaneously formed a hairpin structure, preventing the ThT from intercalating and thus exhibiting a weak fluorescent signal. With an increase in the concentration of Hg (II), the T-base-rich cDNA-9 formed T-Hg(II)-T base pairs with G3MB6, disrupting the original hairpin structure of G3MB6 and allowing the formation of a G3 structure. The structural change enabled ThT to embed within the G3 and excite fluorescence. The fluorescence signal reached its maximum when the concentration of Hg(II) was 300 nM, as the T bases of cDNA-9 in the system had formed T-Hg(II)-T mismatches, completely disrupting the hairpin structure. Further enhancement of the concentration of Hg(II) could not continue to improve the fluorescence signal.

The change in the fluorescence signal (F-F0) under excitation by ThT was linearly correlated with a concentration of Hg(II) of 0–300 nM. The linear fitting equation was y = 1.219x + 13.55, where y stands for F-F0 and x stands for the concentration of Hg(II), with an R^2^ value of 0.9954. The sensor’s limit of detection (LOD) for Hg(II) was 5.32 nM, calculated according to the 3σ/slope criterion. And based on the formula of 10σ/slope, the LOQ value of the sensor was 17.72 nM. This indicated that the G3MB6/ThT sensor could quantitatively detect Hg(II) within the concentration range of 17.72–300 nM with high sensitivity.

To investigate the selectivity of the G3MB6/ThT sensor for detecting Hg(II), we introduced 600 nM Hg(II) and 3 µM of other metal ions, including Ni(II), Mg(II), Fe(II), Fe(III), K(I), Co(II), Mn(II), Ca(II), and Cu(II), as interfering cations. The change in the fluorescence values (F-F0) is illustrated in Figure 5B. The results demonstrated that only Hg(II) generated a significant fluorescent signal by embedding ThT in the G3 structure. Even when other metal ions were introduced at much higher concentrations than Hg(II), they did not produce notable fluorescence signals. This indicated that the G3MB6/ThT sensor possesses high specificity for detecting Hg(II), as the detection method relies on forming T-Hg(II)-T mismatched structures. Furthermore, when Hg(II) was present along with other metal ions, the fluorescence intensity was almost identical to that observed with Hg(II) alone (Figure S5), further confirming that this method has high selectivity for detecting Hg(II).

Additionally, we compared this assay with other recently developed sensors for detecting Hg(II) based on non-labeling methods, focusing on the linear range and LODs. Table 1 summarizes the linear range and LOD of detecting Hg(II) in various systems, illustrating their sensitivity and practicality. Compared with other strategies, this method exhibited a favorable LOD, detection efficiency, and sensitivity in its linear range. These characteristics highlight its feasibility for detecting Hg(II).

### 2.5. Analysis of Hg(II) in Tap Water and Milk

To evaluate the accuracy and practicality of the newly developed G3MB6/ThT sensor for the determination of Hg(II) in real-world samples, G3MB6 and cDNA-9 were utilized to assess the levels of Hg(II) in milk and tap water samples, incorporating 20, 100, and 250 nM Hg(II) solutions. Upon excitation at 430 nm, the fluorescence intensities at 505 nm were recorded, and the concentrations of Hg(II) were derived using the equation y = 1.219x + 13.55, where y denotes F-F0 and x denotes the concentration of Hg(II). The recovery of Hg(II) in milk samples ranged from 100.30% to 103.20%, as shown in Table 2. Moreover, the recovery of Hg(II) in tap water was calculated, as detailed in Table 2, and was from 101.67% to 103.75%. The detection system’s relative standard deviations (RSDs) were consistently lower than 5% (n = 3), attesting to its high accuracy. These findings underscore the efficacy and precision of the detection method to identify the concentration of Hg(II) in real-world samples, thereby establishing its utility as a reliable tool for environmental and food safety monitoring.

## 3. Materials and Methods

### 3.1. Chemicals and Apparatus

All oligonucleotides were ordered from Sangon Biotech Co., Ltd. (Shanghai, China) and prepared for the stock solution at a concentration of 100 μM. The sequences of different oligonucleotides are displayed in Table S1. The standard 1000 μg/mL Hg(II) solution was ordered from Aoke Biology Research Co., Ltd. (Beijing, China). All metal salts, such as NaCl, KCl, MgCl_2_, CaCl_2_, CoCl_2_, FeCl_3_, FeCl_2_, NiCl_2_, MnCl_2_, CuCl_2,_ and tris(hydroxymethyl)aminomethane, were obtained from Macklin (Shanghai, China). All the solutions were dissolved with ddH_2_O; the resistivity was 15–18.2 MΩ.cm. ThT from Macklin Biochemical Co., Ltd. (Shanghai, China) was also prepared for 100 μM with dimethyl sulfoxide (DMSO) and dd H_2_O (1:1). All solutions were stored at 4 °C.

The intensity of fluorescence was assessed using an F-4500 fluorescence spectrometer (Hitachi, Tokyo, Japan). Under an excitation wavelength of 430 nm, the emission spectra were measured from 450 nm to 650 nm, recording the maximum emission (*Em_max_*) peak at 505 nm. The excitation and emission slit widths of F-4500 were both set at 5 nm. The TMP’s voltage was maintained at 700 V for the optimal detection condition.

### 3.2. Fluorescence of the G-Triplex with the Adjacent dsDNA

For this assay, 1.25 μL of the DNA probe (G3MB1, 20 μM) and 1.5 μL of the complementary DNA strand (cDNA-1, 20 μM) were added to an Eppendorf tube (0.5 mL), then diluted with a buffer to 200 μL, and their final concentrations were 125 nM and 150 nM. To optimize the experimental conditions, the intensity of fluorescence of ThT at 505 nm was examined under different incubation conditions, such as the final concentration of ThT (2–8 μM), the reaction time between G3MB1 and its complementary cDNA-1 (2–360 min), the 50 mM Tris-HCl buffer (50 mM KCl, 100 mM NaCl; pH 7–8.5, the concentration of salts in the 50 mM Tris-HCl buffer (Na(I): 0–200 mM; Mg(II): 0–50 mM), the reaction temperature (25–60 °C), and the reaction time of ThT (1–30 min).

The incubation mixture in an Eppendorf tube (0.5 mL) was composed of a DNA probe (G3MBn, n = 1–5) and complementary DNA strands (cDNAn, n = 1–8), which were diluted with a 50 mM Tris-HCl buffer (50 mM KCl, 100 mM NaCl, and 20 mM MgCl_2_, pH = 7.6) to final concentrations of 125 nM and 150 nM. The mixture was mixed entirely in a shaker and then incubated at 37 °C for 60 min. The concentration of the DNA probe and cDNA was 1:1.2. Afterward, 8 μL ThT (100 μM) was added to the abovementioned incubated solution to reach a total volume of 200 μL. Each sample was incubated at room temperature (25 °C) for 5 min. For optimization of G3MBn (n = 1–5), we studied the effects of the number of gap bases between the ds-DNA and the G-triplex from 0 to 3; the length of the hairpin stem formed by the probe itself from 4, 6, and 8 bases; and different structures of the G-triplex.

### 3.3. Fluorescence of Hg(II)

For this, 125 nM DNA probe (G3MB6), 150 nM complementary DNA strands (cDNA-9), and a 600 nM Hg(II) solution were added to an Eppendorf tube (0.5 mL). To optimize the experimental conditions, the intensity of the fluorescence of ThT at 505 nm was examined under different incubation conditions, such as G3MB6, cDNA-9, and Hg(II); reaction times (5, 30, 60, 240, and 720 min); and reaction temperatures (4, 25, 30, and 37 °C).

The reaction solution was prepared as mentioned above. A Tris-HCl (50 mM) buffer containing 50 mM KCl, 100 mM NaCl, and 20 mM MgCl_2_ (pH = 7.6) was adjusted to 192 μL and mixed with the reactants thoroughly, then incubated in a shaker at 25 °C for 60 min. The concentration of G3MB6 and cDNA-9 was 1:1.2. Subsequently, 100 μM ThT (8 μL) was added to each sample to obtain a final volume of 200 μL and reacted at 25 °C for 5 min. For the sensitivity of detecting Hg(II) in the system, the final concentrations of Hg(II) solution were 0, 2, 4, 6, 8, 10, 20, 40, 60, 80, 100, 150, 200, 250, 300, and 350 nM. In the selectivity experiment, interference ions Ni(II), Co(II), Mg(II), K(I), Fe(III), Fe(II), Mn(II), Ca(II), and Cu(II) in a final concentration of 3 μM were used as interfering substances in the 600 nM Hg (II) solution. When the factors were not the experimental subjects, they were kept under the optimal conditions for detection. Each sample underwent analysis at least three times.

### 3.4. Detection of Hg(II) in Natural Samples

To evaluate the performance of the biosensor detection system in natural samples, G3MB6 and cDNA-9 were used to detect concentrations of Hg(II) in water and milk, measuring the recovery rates of Hg(II). Thus, the concentrations of Hg(II) were 20, 100, and 250 nM. Each group of samples underwent analysis at least three times.

## 4. Conclusions

Capitalizing on the phenomenon of G3/ThT’s enhancement of fluorescence, catalyzed by the adjacent dsDNA situated at the 5′ terminus of the G3, our investigation unveiled several critical findings. Firstly, we observed that a four-base stem within the hairpin structure, crafted by single strands, effectively curtailed the formation of G3, affording a notable advantage in the reaction time compared with longer stem lengths. Simultaneously, the emergence of dsDNA at the 5′ terminus orchestrated the alignment of the G3 moiety into a parallel configuration, thereby amplifying the affinity of ThT towards the G-3 and yielding a conspicuous fluorescence signal at 505 nm, coupled with the highest F/F0 ratio. We identified the optimal configuration, indicating that two or three bases between the G3 and dsDNA profoundly influenced ThT’s integration into the G3 structure. Moreover, our study pioneered a novel Hg(II) detection strategy based on G3/ThT. Leveraging the interaction between G3MB6 and its complementary strand, cDNA-9, when Hg(II) existed, we facilitated the formation of a T-Hg(II)-T complex, mimicking TA base pairs. The molecular rearrangement effectively exposed the G3 structure at the 3′ end of G3MB6, thereby harnessing the potential enhancement of the fluorescence of G3/ThT. Subsequent sensitivity and selectivity analyses underscored the system’s remarkable efficacy and specificity for detecting Hg(II). Applying the G3MB6/ThT sensor to monitor Hg(II) levels in milk and tap water samples yielded promising outcomes, with average recovery rates confirming successful detection. The G3MB6/ThT sensor that was developed demonstrated remarkable precision in identifying Hg(II) in environmental samples. Its rapidity, simplicity, and accuracy position it as a valuable asset for assessing water quality and environmental conditions, thus promising impactful practical applications.

## Figures and Tables

**Table 1:** Comparison with the label-free methods for detecting Mercury (Hg(II)) by fluorescence (“on–off” or “off–on”).

Intercalator	Linear Range (μM)	LOD (nM)	References
DAPI + NMM	0–20	300	[27]
DAPI	0–1.4	2.87	[16]
NMM	0–1	18.6	[28]
Berberine	0–10	7.7	[29]
Genefinder	0–0.3	2.5	[30]
ThT	0–4	20	[31]
ThT	0–0.3	5.32	This work

**Table 2:** Recovery rates of concentrations of Hg(II) in milk and tap water (n = 3).

Sample	Group	Added (nM)	Repeats	Found (nM)	Recovery (%)	Avg. (nM)
Milk			1	19.24		
	No.1	20	2	19.24	100.30	20.061
			3	21.70		
			1	102.11		
	No.2	100	2	102.93	103.20	103.203
			3	104.57		
			1	253.08		
	No.3	250	2	255.54	101.12	252.803
			3	249.79		
Water			1	19.24		
	No.1	20	2	20.06	101.67	20.334
			3	21.70		
			1	102.93		
	No.2	100	2	103.75	103.75	103.750
			3	104.57		
			1	254.72		
	No.3	250	2	256.36	102.54	256.359
			3	258.00		

## Data Availability

No new data were generated or analyzed in support of this research.

## Supplementary Materials

The following supporting information can be downloaded at: https://www.mdpi.com/article/10.3390/ijms25158159/s1.
