# Supplementary material for: Comparison of Double-Stranded DNA at the 5′ and 3′ Ends of the G-Triplex and Its Application in the Detection of Hg(II)
Source: Int J Mol Sci. 2024 Jul 26;25(15):8159. doi: 10.3390/ijms25158159 (PMC11311761; doi:10.3390/ijms25158159)
Supplement: Supplementary file 1 [file ijms-25-08159-s001.zip › ijms-3076771-supplementary.pdf]

Comparison of Double-Stranded DNA at the 5′ and 3′ Ends of the G-Triplex and Its Application in the Detection of Hg(II)

Yule Cai †, Ziyi Wu †, Xiangxiang Li, Xingting Hu, Jiamin Wu, Zhengying You * and Jieqiong Qiu *

College of Life Sciences and Medicine, Zhejiang Sci-Tech University, Hangzhou 310018, China

* Correspondence: qiujieqiong@zstu.edu.cn (J.Q.); zyyou@zstu.edu.cn (Z.Y.); Tel.: +86-0571-86843192 (J.Q.)

† These authors contributed equally to this work.

Table S1. The sequences of oligonucleotides involved in this study.

Name	Sequence（5'-3'）
G3MB1	TGCTAAGTCCCGAGCTATATGGGAAGGGAGGG
G3MB2	TGGGAAGGGAGGGATATCGTGCCCTGAATCGT
G3MB3	TGCTACTTCCCGAGCTATATGGGAAGGGAGGG
G3MB4	TGCTATCCCTTCCGCTATATGGGAAGGGAGGG
G3MB5	TGCTACTACCCGAGCTATATGGGTAGGGCGGG
G3MB6	TGCTTAGTCCCTAGCTATATGGGAAGGGAGGG
cDNA-1	AGCTCGGGACTTAGCA
cDNA-2	TAGCTCGGGACTTAGCA
cDNA-3	ATAGCTCGGGACTTAGCA
cDNA-4	TATAGCTCGGGACTTAGCA
cDNA-5	ACGATTCAGGGCACGAT
cDNA-6	TAGCTCGGGAAGTAGCA
cDNA-7	TAGCGGAAGGGATAGCA
cDNA-8	TAGCTCGGGTAGTAGCA
cDNA-9	TAGCTTGGGTCTTTGCA

Figure S1 Fluorescence emission spectra of G3MB1+cDNA-1 with different ThT concentrations and reaction times.

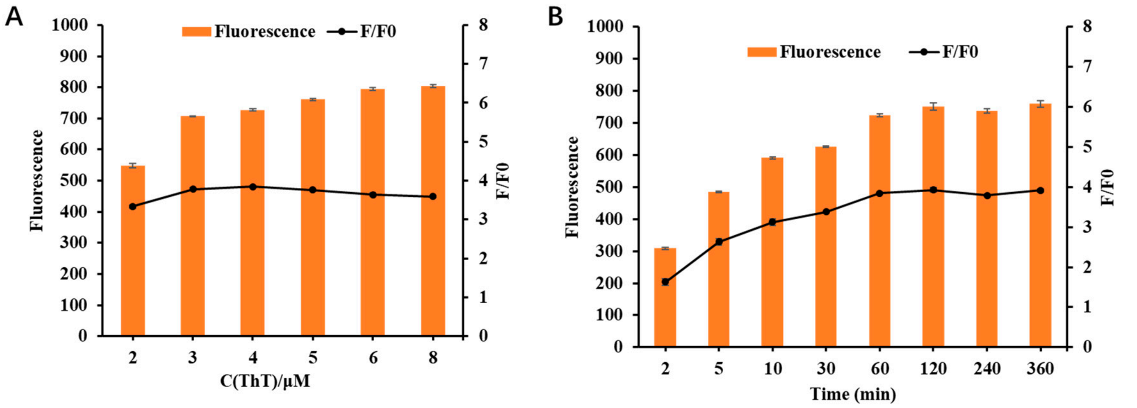

Figure S1. A: 125 nM of G3MB1 and 150 nM of cDNA-1 were reacted in the 50 mM Tris-HCl buffer (pH=7.6) containing 50 mM KCl and 100 mM NaCl at 37 °C for 2 hrs, followed by the addition of ThT at the final concentrations of 2 μM, 3 μM, 4 μM, 5 μM, 6 μM, and 8 μM, respectively, and the reaction was performed at roomtemperature for 30 min, and the fluorescence signal was detected at *Ex* =430 nm, *Em* = 505 nm; B: 125 nM of G3MB1 and 150 nM of cDNA-1 were reacted in 50 mM Tris-HCl, a buffer (pH=7.6) containing 50 mM KCl and 100 mM NaCl at 37 °C for 2 min, 5 min, 10 min, 30 min, 60 min, 120 min, 240 min, and 360 min, followed by the addition of ThT at a concentration of 4 μM, and the reaction was carried out for 30 min at room temperature, and the fluorescence signal was detected at *Ex* = 430 nm, *Em* = 505 nm.

Figure S2 Fluorescence emission spectra of G3MB1+cDNA-1 at different pH values, Na(I) and Mg(II) concentrations.

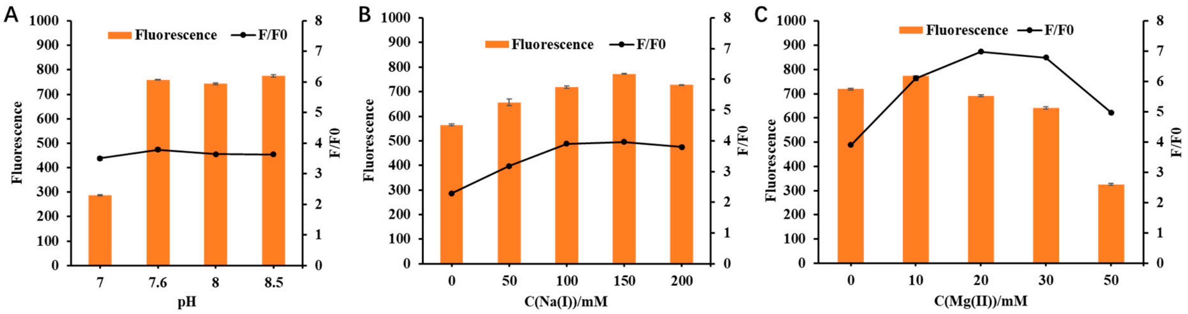

Figure S2. A: 125 nM of G3MB1 and 150 nM of cDNA-1 were reacted in the 50 mM Tris-HCl buffer (pH=7.6) containing 50 mM KCl and 100 mM NaCl, pH 7, 7.6, 8, 8.5, respectively, at 37 °C for 60 min. Subsequently, ThT at a final concentration of 4 μM was added, and the reaction was carried out at room temperature for 30 min, and the fluorescence signal was detected at *Ex* = 430 nm, *Em* = 505 nm; B: 125 nM G3MB1 and 150 nM cDNA-1 were reacted in the 50 mM Tris-HCl buffer (pH 7.6) containing 50 mM KCl and 0, 50, 100, 150, 200 nM NaCl for 60 min at 37 °C, followed by the addition of ThT at a final concentration of 4 μM, and the reaction was carried out for 30 min at room temperature, and the fluorescence signal was detected at *Ex* = 430 nm, *Em* = 505 nm; C: 125 nM G3MB1 and 150 nM cDNA-1 were reacted in the 50 mM Tris-HCl buffer (pH 7.6) containing 50 mM KCl, 100 mM NaCl, and 0, 10, 20, 30, 50 mM $MgCl_2$ for 60 min at 37 °C, followed by the addition of ThT at a concentration of 4 μM for 30 min at room temperature, and fluorescence signal was detected at *Ex* = 430 nm, *Em* = 505 nm.

Figure S3 Fluorescence emission spectra of G3MB1+cDNA-1 at different reaction temperatures and ThT reaction times.

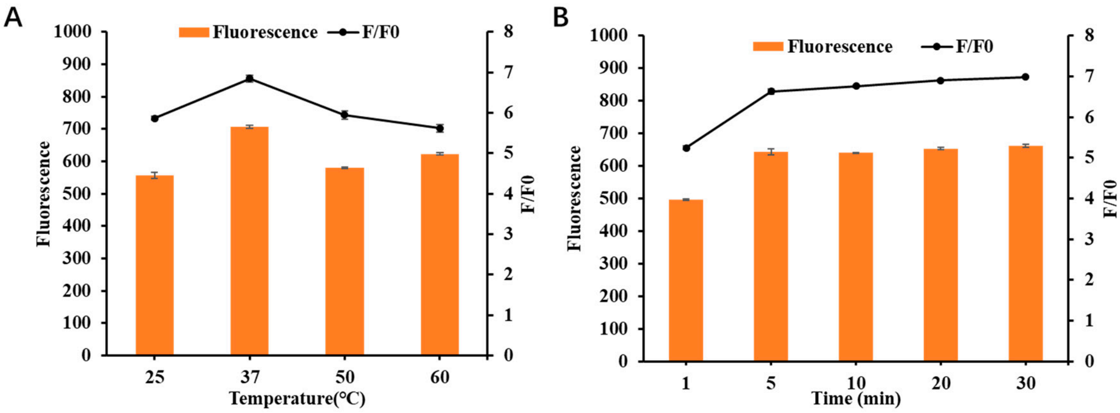

Figure S3. A: 125 nM of G3MB1 and 150 nM of cDNA-1 were reacted in the 50 mM Tris-HCl buffer (pH=7.6) containing 50 mM KCl, 100 mM NaCl, and 20 mM $MgCl_2$, at 25 °C, 37 °C, 50 °C and 60 °C for 60 min. Subsequently, ThT at a final concentration of 4 µM was added, and the reaction was performed at room temperature for 30 min, and the fluorescence signal was detected at *Ex* = 430 nm, *Em* = 505 nm; B: 125 nM G3MB1 and 150 nM cDNA-1 were reacted in the 50 mM-Tris HCl buffer (pH=7.6) containing 50 mM KCl, 100 mM NaCl, and 20 mM $MgCl_2$ for 60 min at 37 °C, followed by the addition of ThT at a final concentration of 4 µM, and the reaction was carried out for 0, 5, 10, 20, 30 min at room temperature, and the fluorescence signal was detected at *Ex* = 430 nm, *Em* = 505 nm.

Figure S4 Relative fluorescence intensity (F/F0) of G3MB6+cDNA-9 with different reaction times and temperatures in the presence of Hg(II).

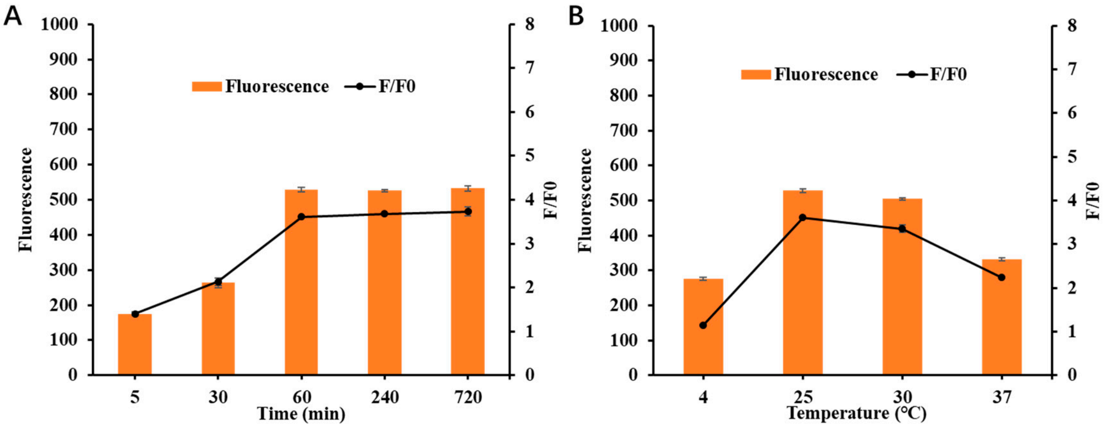

Figure S4. A. Relative fluorescence intensity (F/F0) of G3MB6+cDNA-9 in the presence of Hg(II) solution with different reaction times (5, 30, 60, 240 and 720 min); B. Relative fluorescence intensity (F/F0) of G3MB6+cDNA-9 in the presence of Hg(II) solution with different reaction temperatures (4, 25, 30, 37 °C). Each reaction was conducted in the 50 mM Tris-HCl buffer (50 mM KCl + 100 mM NaCl + 20 mM $MgCl_2$, pH 7.6) by adding 125 nM G3MB6, 150 nM cDNA-9, 4 µM ThT, and 600 nM Hg(II). *Ex* = 430 nm, *Em* = 505 nm.

Figure S5 Fluorescence intensity changes (F-F0) of G3MB6+cDNA-9 in the presence of Hg(II) and other metal ions.

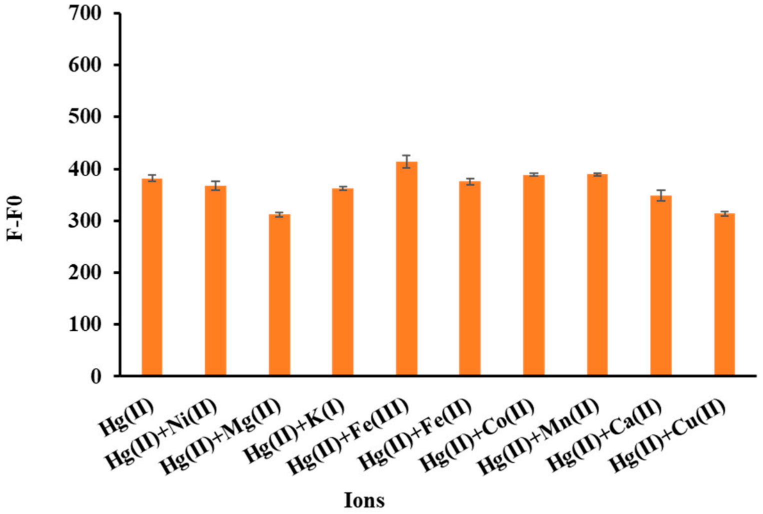

Figure S5. Fluorescence intensity changes (F-F0) of G3MB6 in the presence of Hg(II) mixed with Ni(II), Mg(II), K(I), Fe(III), Fe(II), Co(II), Mn(II), Ca(II), Cu(II). 600 nM Hg(II), 3 µM other metal ions were used, 125 nM G3MB6, 150 nM cDNA-9, and 4 µM ThT were used in a 50 mM Tris-HCl buffer (50 mM KCl + 100 mM NaCl + 20 mM $MgCl_2$, pH 7.6) for each reaction. *Ex* = 430 nm, *Em* = 505 nm.
